# Automatic extraction of cell nuclei from H&E-stained histopathological images

**DOI:** 10.1117/1.JMI.4.2.027502

**Published:** 2017-06-21

**Authors:** Faliu Yi, Junzhou Huang, Lin Yang, Yang Xie, Guanghua Xiao

**Affiliations:** a University of Texas Southwestern Medical Center, Quantitative Biomedical Research Center, Department of Clinical Science, Dallas, Texas, United States; b University of Texas at Arlington, Department of Computer Science and Engineering, Arlington, Texas, United States; c Chinese Academy of Medical Science and Peking Union Medical College, National Cancer Center/Cancer Hospital, Department of Pathology, Chaoyang District, Beijing, China; d University of Texas Southwestern Medical Center, Department of Bioinformatics, Dallas, Texas, United States; e University of Texas Southwestern Medical Center, Harold C. Simmons Comprehensive Cancer Center, Dallas, Texas, United States

**Keywords:** digital pathology, hematoxylin and eosin-stained image, cell nuclei extraction, watershed transform, color deconvolution, image analysis

## Abstract

Extraction of cell nuclei from hematoxylin and eosin (H&E)-stained histopathological images is an essential preprocessing step in computerized image analysis for disease detection, diagnosis, and prognosis. We present an automated cell nuclei segmentation approach that works with H&E-stained images. A color deconvolution algorithm was first applied to the image to get the hematoxylin channel. Using a morphological operation and thresholding technique on the hematoxylin channel image, candidate target nuclei and background regions were detected, which were then used as markers for a marker-controlled watershed transform segmentation algorithm. Moreover, postprocessing was conducted to split the touching nuclei. For each segmented region from the previous steps, the regional maximum value positions were identified as potential nuclei centers. These maximum values were further grouped into k-clusters, and the locations within each cluster were connected with the minimum spanning tree technique. Then, these connected positions were utilized as new markers for a watershed segmentation approach. The final number of nuclei at each region was determined by minimizing an objective function that iterated all of the possible k-values. The proposed method was applied to the pathological images of the tumor tissues from The Cancer Genome Atlas study. Experimental results show that the proposed method can lead to promising results in terms of segmentation accuracy and separation of touching nuclei.

## 1 Introduction

With the advent of fast digital slide scanners, tissue histopathology slides are now able to be digitized and stored in a digital image form that can be repeatedly accessed and examined by pathologists.^1^^–^^5^ In practice, different components of the tissue are dyed with different stains so that the specific tissue components can be differentiated in digital histopathology images, to facilitate visual inspection by pathologists. Hematoxylin and eosin (H&E) staining is a widespread staining protocol and has been widely used in pathological staining. Hematoxylin stains the nuclei in a dark blue color while eosin stains cytoplasm as pink,^5^ which enables morphological feature analysis related to cell nuclei.

Pathological examination, in which a series of H&E-stained histopathological slides are manually examined by pathologists for disease diagnosis, is a time-consuming and labor-intensive task. More importantly, this process is subjective, prone to error, and has large inter- and intraobserver variation. Due to the heterogeneity and morphological complexity of tumors, it is a challenging task even for well-trained pathologists to reach an agreement when diagnosing a tumor sample by visual inspection of H&E-stained images. For example, the reproducibility and consistency of breast cancer grading are poor with a manual analysis method.^5^^–^^7^ Diagnosis results with a traditional visualization scheme may be even less reliable in developing countries because of the persistent shortage of sufficiently trained pathologists. Therefore, it is essential to develop an automated analysis system to improve the efficiency and accuracy of disease diagnosis by digital pathological images.

Automated cell nuclei segmentation is an essential preprocessing step in various automated analysis systems that use digital histopathological images, including cancer classification, recognition, and grading.^5^^–^^10^ In the segmentation step, a digital image is partitioned into multiple parts, in which each part has a similar texture or intensity value.^11^^–^^14^ Segmentation is usually the first and most vital step since the segmentation results directly determine the success of the final analysis. Consequently, a variety of segmentation approaches have been developed. In a broad sense, the segmentation methods can be divided into six categories: thresholding,^11^^,^^15^ region growing,^11^^,^^16^ clustering,^11^^,^^17^ watershed,^11^^,^^18^ active contour model,^19^^,^^20^ and graph cut.^21^^,^^22^ Segmentation of cell nuclei has been attempted using threshold-based methods.^23^^–^^26^ However, these methods may lead to under- and oversegmentation problems due to the variability across images or heterogeneity within the cell nuclei. On the other hand, threshold-based methods are very efficient and have been widely used as an initial step for further processing or in combination with other methods, such as morphological operation^24^^,^^27^^,^^28^ and the watershed transform technique,^29^ to achieve the final segmentation results. In Ref. 30, the region growing algorithm is used to extract the cell nuclei. The proposed methods in Ref. 30 would still result in an oversegmentation problem when heterogeneity appeared in the cell nuclei, which is a common situation in H&E-stained images. Even though the region growing-based method in Ref. 31 can solve the heterogeneity problem to some extent by using multiple scale images, it cannot handle the touching and overlapping cell nuclei. In Refs. 32–37, clustering-based methods were applied to the nuclei segmentation, but it was found that the clustering method is sensitive to the intensity variations within the nuclei. Furthermore, it is challenging to define the number of clusters when the image is complex. Therefore, the clustering algorithm is sometimes used as a preprocessing step for further nuclei segmentation.^38^ In Refs. 33, 39–42, the cell nuclei were identified with the watershed algorithm, and the segmented regions were further merged with other methods, such as region growing and graph cut. To reduce the oversegmentation problem, the marker-controlled watershed algorithm is used in cell and cell nuclei segmentation.^5^^,^^29^^,^^43^^,^^44^ The marker-controlled watershed technique is also widely used to separate touching or overlapping nuclei as a postprocessing step.^45^^–^^47^ The difficulty for this algorithm is how to better identify these markers in nuclei images. In Ref. 47, the markers are determined with distance transform,^11^ whereas in Ref. 5, the markers are estimated with gradient-weight distance transform.^5^ In Ref. 48, the markers are decided with H-minima transform.^48^^,^^49^ However, these marker detection methods sometimes make one target have more than one marker because they are unable to derive the number of targets within a region. Therefore, they still produce an oversegmentation problem. In Refs. 50–52, the active contour models are used to segment the cell nuclei. However, these methods still produce an oversegmentation problem when high variation exists within the nuclei and cannot separate the touching or overlapping nuclei without further processing. Moreover, these methods are sensitive to initialization and other artifacts present in the tissue image. They are also restricted in computational efficiency. In Refs. 53 and 54, methods based on graph cut are used to segment the cell nuclei. However, they cannot split the touching and overlapping nuclei. In Ref. 42, the two-stage graph cut is applied to segment the overlapping nuclei, but it is not easy to assign the corresponding weight. In Ref. 54, graph cut combined with the α-expansion algorithm^54^^,^^55^ is utilized for nuclei segmentation. It is computationally expensive and loses the global optimal minimum value of the graph cut. In Ref. 56, nuclei segmentation is achieved by incorporating nuclei shape information into the graph cut. But it considers only the healthy nuclei, and this makes its application limited.

**Fig. 1:**
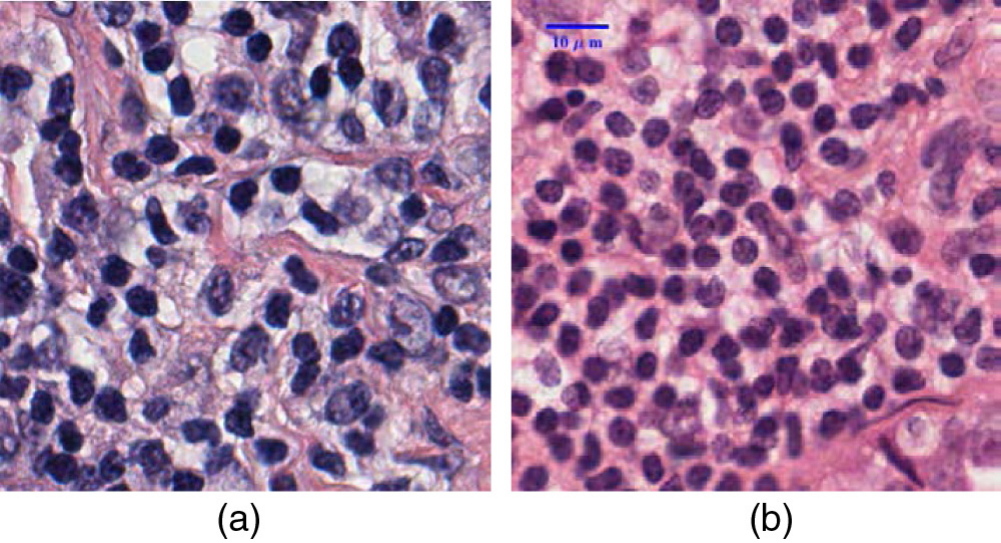
Two H&E-stained histopathological images (magnification: 40×).

In summary, there is no single method that can handle all the segmentation problems well. For automated cell nuclei segmentation for H&E-stained histopathology images, there are three challenges.^48^^,^^57^ First, there is large variation among H&E-stained images, which is probably caused by the process of slide preparation and image acquisition. Second, the intensities of the background regions (nonnuclei areas) in H&E-stained images are uneven, which complicate the separation of nuclei and nonnuclei. Third, a three-dimensional structure of tumor tissues is captured as a two-dimensional histopathological image, in which cell nuclei are often “touching” and “overlapping” with each other, which makes it difficult to separate the individual cell nuclei. For example, two H&E-stained histopathological images are shown in Fig. 1. A robust cell nuclei segmentation algorithm is needed to overcome the aforementioned problems.

In this study, we aim to develop a fully automatic method for nuclei segmentation in H&E-stained histopathology images. The color deconvolution technique, thresholding, and morphological operations are applied as preprocessing steps for nuclei segmentation so as to make the method robust to the variation and heterogeneity existing in nuclei images. The marker-controlled watershed is used to handle the touching nuclei problem while k-means combined with an objective function is utilized to find the appropriate number of nuclei within overlapping regions. The paper is organized as follows: in Sec. 2, the proposed algorithm is presented in detail. In Sec. 3, we show the experimental results. In Sec. 4, we give the conclusions and future work.

## 2 Methodology

**Fig. 2:**
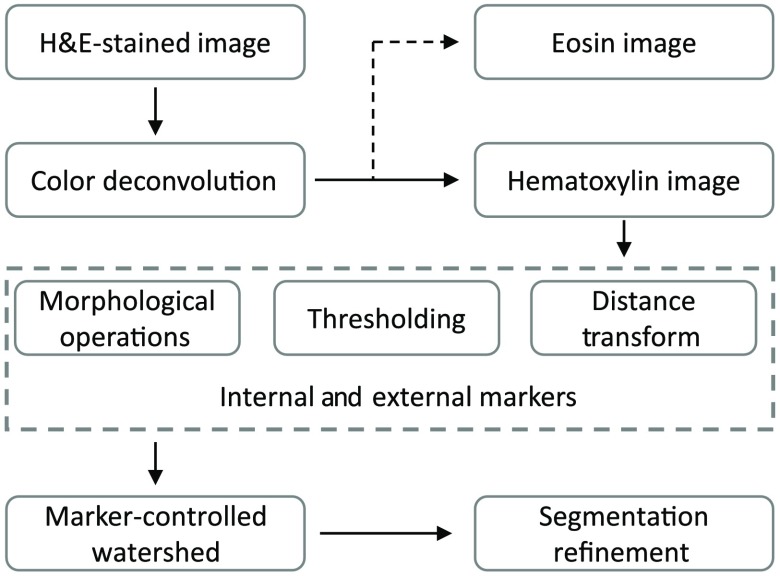
Flow diagram of the proposed segmentation algorithm.

The method begins with a color deconvolution algorithm that separates the H&E-stained histopathology image into H&E channels. Then, morphological operations and thresholding techniques are applied to the hematoxylin channel so that the markers are determined for the use of nuclei segmentation with the marker-controlled watershed transform algorithm. Finally, the segmentation results are refined with the marker-controlled watershed approach again by minimizing an objective function that can estimate the number of overlapping nuclei in the segmented regions. The flow diagram of our proposed method is shown in Fig. 2.

### 2.1 Color Deconvolution

The color deconvolution framework was proposed by Ruifrok and Johnston^58^ in 2001 and has been successfully applied in histopathology images.^5^ The color deconvolution technique is based on the fact that the imaging process can be simulated with the Lambert–Beer law.^58^ Consequently, the relationship between the RGB color space Ω and a new color space € defined by the stains can be expressed as

**Fig. 3:**
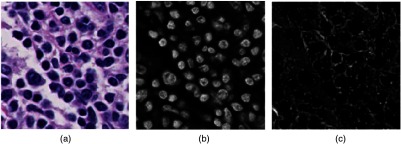
Color deconvolution: (a) H&E-stained image, (b) hematoxylin channel image, and (c) eosin channel image.

Ω=exp(−M€), (1)

where M is the stain matrix or convolution matrix. Therefore, the intensity referring to the stain concentration in the new color space € is derived as

$$€=M^{-1}£,$$ (2)

where $M^{-1}$ is the inverse of stain matrix M and £=−log(Ω) represents the optical density, which has combined the information on the absorbance and concentration of stains.^58^ Therefore, the amount of each stain in color space € can be achieved once the stain matrix or convolution matrix M is estimated. Even though many methods were proposed to estimate the stain matrix, the method presented in Ref. 59, which has shown better performance and is insensitive to imaging conditions, is applied in our proposed nuclei segmentation method. An example of color deconvolution from RGB space to H&E space is shown in Fig. 3. It can be visually seen that the adopted algorithm is robust to separating the RGB image into the H&E image. All the subsequent processing is conducted on the hematoxylin channel image since the cell nuclei in the tissue section are dyed with hematoxylin.

### 2.2 Morphological Operations

Even though the color deconvolution algorithm can separate the image from RGB space into H&E space, the hematoxylin image still has an intensity variation problem within the cell nuclei. Fortunately, the morphological operations can remove unnecessary structures within the nuclei and make the nuclei region much smoother. The two main morphological operations applied to the hematoxylin image are opening by reconstruction and closing by reconstruction.^11^ Opening by reconstruction is a morphological transform involving morphological erosion^11^ followed by morphological reconstruction.^11^ It can filter out the unconnected bright targets that are smaller than the structuring element and preserve the shape of image objects that are bigger than the structuring element. Conversely, closing by reconstruction is defined as the morphological dilation operation^11^ followed by the morphological reconstruction operation.^11^ This operation can remove unconnected dark objects smaller than the structuring element in the cell nuclei while still leaving the background unchanged. As described in Ref. 5, the differing sizes of the structuring element^11^ used in morphological operations will result in varied segmentation results. It is also claimed that the size of the structuring element should be selected according to the size of the nuclei and the resolution of the H&E-stained image.^5^ In this study, a disk-shaped structuring element with a radius of ∼7 is used while the magnification of the histopathological slide image is 40×. We also experimentally verify that our algorithm is not very sensitive to the size of the structuring element when the radius of the structuring element is smaller than 8 in this step. Furthermore, the morphological filling operation^11^ is applied to the image after applying opening by reconstruction and closing by reconstruction, to make the segmentation result less sensitive to the size of the structuring element.

### 2.3 Thresholding

**Fig. 4:**
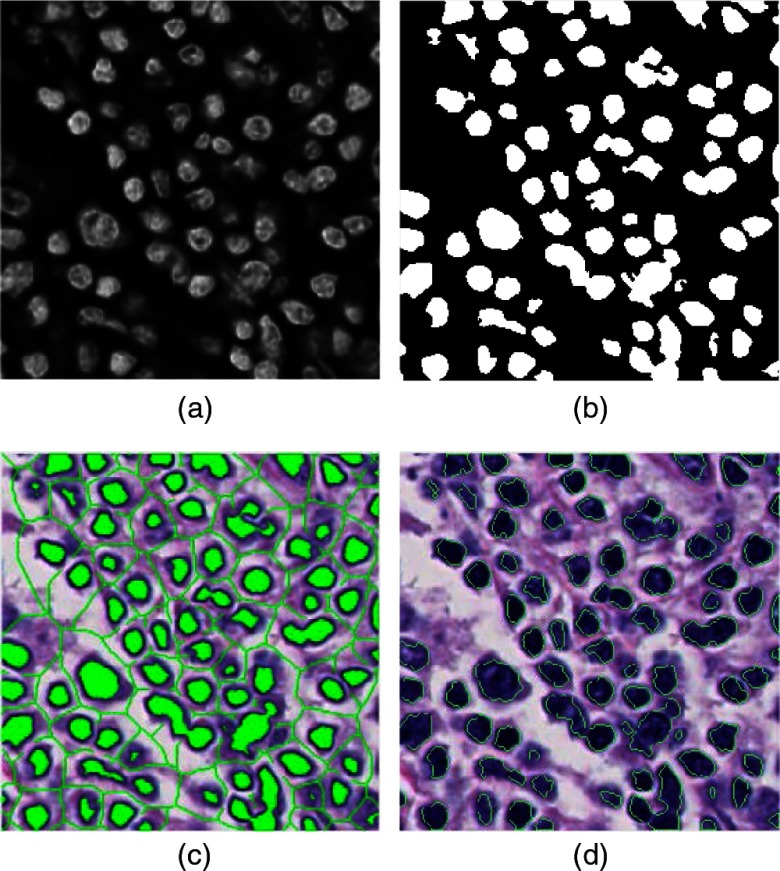
Segmentation processes: (a) hematoxylin channel image, (b) thresholding, (c) markers (green color), and (d) segmentation results.

After a series of morphological operations to the hematoxylin channel, the cell nuclei tend to be flat and the difference between nuclei and background is enlarged. That is, the image starts to consist of two classes (foreground and background), and the pixels in the image follow a bimodal histogram. Consequently, an automated thresholding technique will work well to briefly detect the cell nuclei. On the other hand, if a fixed threshold is used to identify the nuclei in the thresholding segmentation, it will fail when different images with high variation need to be processed. In this paper, Otsu’s method^11^ to automatically find the threshold value is applied to briefly segment the cell nuclei. Otsu’s algorithm exhaustively searches for the threshold that can minimize the intraclass variance, which is given as^11^

$$\sigma_{\omega }^{2}(T)=\omega_{1}(T)\sigma_{1}^{2}(T)+\omega_{2}(T)\sigma_{2}^{2}(T),$$ (3)

where $\sigma_{i}^{2}$ (1≤i≤2) are the variances of the two classes and weights $\omega_{i}$ are the probabilities of the two classes separated by a threshold T. The weights $\omega_{1}(T)$ and $\omega_{2}(T)$ can be further obtained with the following equations:

$$\omega_{1}(T)=\overset{T}{\underset{i=1}{\sum }}P(i)\;\text{and}\;\omega_{2}(T)=\overset{I}{\underset{i=T+1}{\sum }}P(i),$$ (4)

where I is the maximum intensity value and P(i) is the probability of intensity value i occurred in the image. An example of applying Otsu’s method to a morphologically processed image is shown in Fig. 4(b).

### 2.4 Marker-Controlled Watershed Transform Segmentation

For marker-controlled watershed segmentation, the main step is to appropriately identify the markers, which consist of internal and external markers.^11^^–^^13^ The internal markers represent the cell nuclei that we are looking for, while the external markers represent the background regions around all of the cell nuclei. The external markers should be a connected component in the image. When the markers are determined, the watershed transform can find the peaks or watersheds between the internal and external markers based on the magnitude gradient hematoxylin image.

After applying the thresholding operation with Otsu’s method to the morphologically operated hematoxylin image, the boundaries of some nuclei in the resulting binary image would appear irregular and have some protrusions. Therefore, the morphological opening operation, which is a morphological erosion followed by a dilation operation,^11^ is used to make the boundary smooth. The disk-shaped structuring element with radius 3 is utilized for the morphological opening, which can smooth the boundary and remove small protrusions at the same time. To mark the internal markers referring to cell nuclei, the distance transform algorithm^11^ is first applied to the smoothed binary image. To be specific, the distance transform of a foreground pixel p (location with 1 value in the binary image) is calculated as^11^

$$D(p)=\min_{q\in B}\;d(p,q),\;p\in F,$$ (5)

where pixel p belongs to the set of background pixels B (locations with zero value in the binary image), F is the set of foreground pixels, and d(p,q) means the distance between pixel p and pixel q. Here, the Euclidean distance is adopted while other distance metrics, such as chessboard, cityblock, and quasi-Euclidean, can be also used.^60^ With this distance transform, the values would be zero at the background regions and the relatively high values would tend to be at the locations of the centers of the cell nuclei. Then, the internal markers are obtained by using H-maxima transform^48^ with a threshold value of 3 on the distance-transformed image. The H-maxima transform can suppress all maxima in intensity images with an intensity value smaller than the threshold value to be zero, and it can make the values of other maxima locations to be the threshold value. Therefore, the locations having a threshold value in the H-maxima transform are regarded as the foreground and used as the internal markers. The distance transform and H-maxima transform can make the internal markers keep the shape of the cell nuclei. Since the threshold value used in the H-maxima transform is the small value 3, it can only separate the slightly touching cell nuclei. However, we focus on nuclei separation in the segmentation refinement part and try to make the preceding processes as flexible as possible. The small threshold value in H-maxima transform would make it robust enough to avoid losing some small cell nuclei. On the other hand, the locations with zero values in H-maxima transform are taken as the background, and the morphological skeleton^11^ of the background is viewed as external markers. The morphological skeleton of a connected region I is expressed in terms of erosions and openings and can be given as^11^

$$S(I)={⋃}_{K=0}^{K}\{(I⊖kB)-[(I⊖kB)\circ B]\},$$ (6)

where B is a structuring element, (I⊖kB) means k-successive erosions of region I while K is the last iterative step before I erodes to empty, and ∘ indicates the morphological opening operation.^11^

Once the internal and external markers are determined, they are combined as final markers and used to modify the magnitude gradient hematoxylin image that can be achieved with Sobel detection or morphological operation methods.^11^ The gradient image is adjusted by the use of the minima imposition technique^11^ so that the regional minima in the gradient image only occur at the locations that have markers. Applying the minima imposition algorithm to the gradient hematoxylin image, $I_{g}$ with final markers M is described as follows:^11^

$$I_{g}^{\prime }=R_{\left[(I_{g}+1)\wedge M\right]}^{\epsilon }(M),$$ (7)

where $R_{A}^{\epsilon }(B)$ means the morphological erosion reconstruction of B from A and ∧; stands for the point-wise minimum between ($I_{g}+1$) and M. Therefore, the oversegmentation problem is reduced when the watershed transform technique is applied to the modified gradient hematoxylin image. An example of the H&E-stained image labeled with markers (internal and external markers) and the segmentation result from the marker-controlled watershed transform algorithm are shown in Figs. 4(c) and 4(d).

### 2.5 Segmentation Refinement

Even though the preceding processing steps can get coarse segmentation results quickly and separate the slightly touching cell nuclei, some overlapping nuclei cannot be divided, and, thus, a refinement process is necessary. The main method applied for splitting the overlapping nuclei is also a marker-controlled watershed transform algorithm, and the key point is how to determine the number of cell nuclei in a connected region. Other algorithms used in this section consist of gradient-weighted distance transform,^61^ k-means,^32^ and minimum spanning tree.^62^

**Fig. 5:**
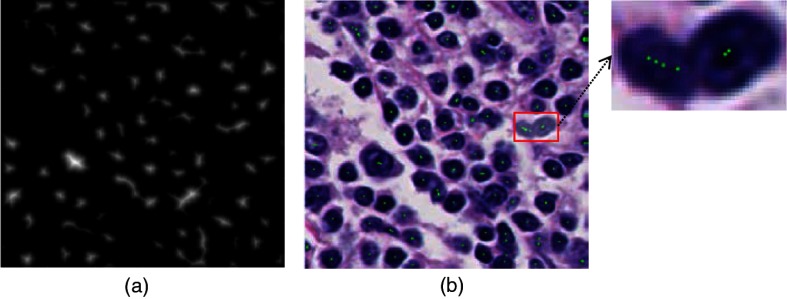
Regional maxima detection: (a) gradient-weighted distance transform to the mask of segmented image and (b) regional maxima (marked in green dot).

In this refinement step, each region, ϑ, in the segmented hematoxylin image from the previous result is evaluated separately. First, each region, ϑ, is conducted with gradient-weighted distance transform.^61^ Gradient-weighted distance transform, as its name reveals, combines the image gradient with the spatial distance information. It can be mathematically expressed as^11^

$$D_{g}=D\times \exp(1-\frac{G-G_{\min}}{G_{\max}-G_{\min}}),$$ (8)

where D is the distance transform map, G is the gradient transform map, and $G_{\min}$ and $G_{\max}$ are the minimum and maximum values in the gradient map G, respectively. It can be noted that the $D_{g}$ value tends to be high at positions closer to the target center and locations with smaller gradient values. On the other hand, the $D_{g}$ value becomes smaller at the object boundary and at positions with big gradient values. The gradient-weighted distance transform is suited for providing the separation cue because the pixels with larger gradient values are more likely to be located at the nuclei boundary or at the boundary among overlapping cell nuclei. Then, the regional maxima in gradient-weighted distance transform image $D_{g}$ are detected. Supposing a total n regional maxima are achieved in this step, it means there may, at most, be n objects in this region because more than one regional maxima can be detected on some targets due to the irregularity of the target shape. Some regions with multiple regional maxima are presented in Fig. 5.

**Fig. 6:**
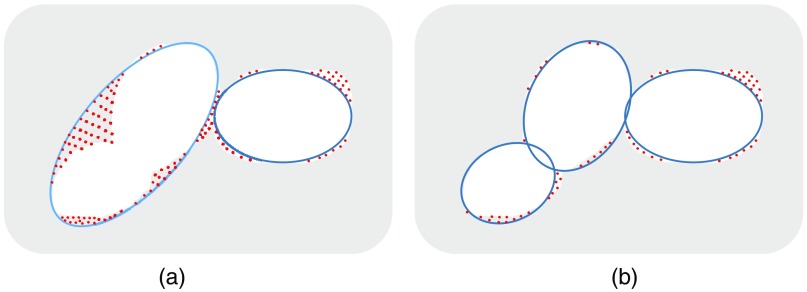
Illustration of objective function in Eq. (11). [The area under red dot mark is the value of Eq. (11), the white object is cells and the fitted ellipse is marked in blue.]

Then, the k-means algorithm is applied to the n regional maxima points. k-means is a clustering technique that aims to minimize the within-cluster sum of squares defined as

$$O=\overset{k}{\underset{j=1}{\sum }}\overset{m}{\underset{i=1}{\sum }}{\left\|x_{i}^{\left(j\right)}-c_{j}\right\|}^{2},$$ (9)

where k is the number of clusters, m is the number of data points in the i’th cluster, $x_{i}^{\left(j\right)}$ is the i’th data point in the j’th class, $c_{j}$ represents the mean of points in the j’th group, and $\left\|x_{i}^{\left(j\right)}-c_{j}\right\|$ is the Euclidean distance between $x_{i}^{\left(j\right)}$ and $c_{j}$. To use the k-means algorithm, the number of clusters should be defined in advance. Here, we know that the number of clusters (number of targets in that region) could be any number between 1 and n. As a result, we applied the k-means approach to the n regional maxima points by varying the number of clusters from 1 to n, respectively. Then, the data points within each cluster are connected by using a minimum spanning tree scheme^62^ and are regarded as the internal markers. We utilize the connected maxima points but not the cluster centroid point as the markers because the cluster centroids may be distant from the maxima points, whereas the minimum spanning tree can connect all the elements within the cluster together with the minimal total weighting for its edges. Once the internal markers are determined, the results of morphological erosion to the complement of region ϑ using the structuring element with radius 3 are used as external markers. Then, the marker-controlled watershed transform algorithm is applied to the gradient hematoxylin image modified with the internal and external markers using the minima imposition technique. Consequently, we can get k segmented regions within region ϑ while each subregion is modeled as an ellipse that is represented as

$$\frac{{\left[(x-c_{x})\cos(\alpha )+(y-c_{y})\cos(\alpha )\right]}^{2}}{a^{2}}+\frac{{\left[(x-c_{x})\sin(\alpha )-(y-c_{y})\cos(\alpha )\right]}^{2}}{b^{2}}=1,$$ (10)

where a and b are the major and minor axis, respectively, $c_{x}$ and $c_{y}$ are the center points, and α is the rotation angle between the x-axis and the major axis. All these ellipse parameters are measured by analyzing the connected component in the previously segmented image resulting from region ϑ. Still, the potential number of touching objects in region ϑ is unknown up to now, and we have developed an objective function to estimate the target number based on the assumption that each object tends to be an elliptical shape. The objective function is expressed as

$$\xi (K)=\overset{K}{\underset{i=1}{\sum }}\overset{J}{\underset{j=1}{\sum }}|P_{i}^{j}\notin \vartheta |+\overset{M}{\underset{m=1}{\sum }}|P_{\vartheta }^{m}\notin {⋃}_{i=1}^{K}E_{i}|,$$ (11)

where $P_{i}^{j}$ is the j’th point on the i’th fitted ellipse, $P_{\vartheta }^{m}$ denotes the m’th point on region ϑ, $E_{i}$ represents the i’th fitted ellipse, K means the number of clusters, J is the total number of points within the i’th ellipse, and M is the total number of points within region ϑ. $\left|P_{i}^{j}\notin \vartheta \right|$ equals 1 if $P_{i}^{j}\notin \vartheta$. Otherwise, it is 0. The definition of $\left|P_{\vartheta }^{m}\notin {⋃}_{i=1}^{K}E_{i}\right|$ is similar to $\left|P_{i}^{j}\notin \vartheta \right|$. The illustration of the above equation is given in Fig. 6.

Therefore, the potential number of targets in region ϑ is estimated by choosing the cluster number K that achieves the minimum ξ value, and the corresponding segmentation results are used as the final ones for that region. All other regions are analyzed with a similar procedure so that the overlapping objects are appropriately separated. The whole procedure of this segmentation refinement part is given in Algorithm 1

**Algorithm 1:** Procedure of segmentation refinement.

1: Input previous segmentation image obtained from Sec. 2.4.
2: Label all the regions (suppose a total of M connected regions).
3: Perform gradient-weight distance transform.
4: **For**i**from** 1 **to**M
5: Extract the i’th region.
6: Erode the complement of the i’th region and use the resulting image as external markers.
7: Detect the regional maxima within the i’th region (suppose total N maxima in the i’th region).
8: **For**j**from** 1 **to**N
9: Apply k-means scheme to the N maxima while cluster number is set to be j.
10: Connect cluster members with minimum spanning tree technique and use the connected points as internal markers.
11: Modify gradient hematoxylin image with the above markers (internal+external markers).
12: Apply watershed transform algorithm to modified gradient hematoxylin image.
13: Fit each segmented region with an ellipse shape.
14: Measure ξ value with Eq. (11).
15: **End**
16: Choose j’th segmentation result as the separation result of i’th region where j achieves the minimum ξ value in the above loop.
17:**End**

It should be noted from Algorithm 1 that the iterations within the inner loop are independently processed and the iterations within the outer loop are also independently executed. Therefore, the segmentation refinement steps are suitable for parallel computing. Either the outer or inner loop can be designed as a kernel function on a device [graphics processing unit (GPU)] and runs on a multicore GPU in parallel.^63^ The data resulting from lines 2, 3, 5, 6, and 7 are visited commonly by each iteration, and it is better to load them into the shared memory on GPU to speed-up the data access if the memory size will allow it. The kernel function from the outer loop can even call the kernel function from the inner loop with a Compute Unified Device Architecture dynamic parallelism technique.^63^ However, when the image is very small and only a few regions are detected, such as when the total iteration number in the loop is much smaller than the number of cores on the GPU, the speed-up would be limited because there is a data transfer latency between the central processing unit (CPU) and GPU.

## 3 Experimental Results

In this section, 10 H&E-stained histopathological slide images with lung cancer were randomly selected from The Cancer Genome Atlas (TCGA) dataset. All the slide images we studied were measured based on the tissue of lung cancer and checked by an experienced pathologist, whereas slide images of low quality, such as those containing severe artifacts, were excluded from our research. These slide images in TCGA were obtained using the whole slide scanner at a magnification of 40× with a resolution of 0.25  μm/pixel or 20× with a resolution of 0.50  μm/pixel. In this study, we only used slide images with a resolution of 0.25  μm/pixel. However, the proposed nuclei extraction approach was also performed on slide images with a resolution of 0.50  μm/pixel by either resizing those images or reducing the size of the structuring element used in the morphological operation in our algorithm. From each slide image, two images with sizes of 350×350 and having an average of 70 cell nuclei were randomly selected from a tumor region labeled by a pathologist while analyzing the nontumor region in the slide images was beyond the scope of this paper. Then, the nuclei extraction process was conducted on these images. The segmentation procedure was implemented in MATLAB^®^ R2015b and was conducted on a desktop personal computer with a 3.40-GHz Intel Core i7-4770 processor.

**Fig. 7:**
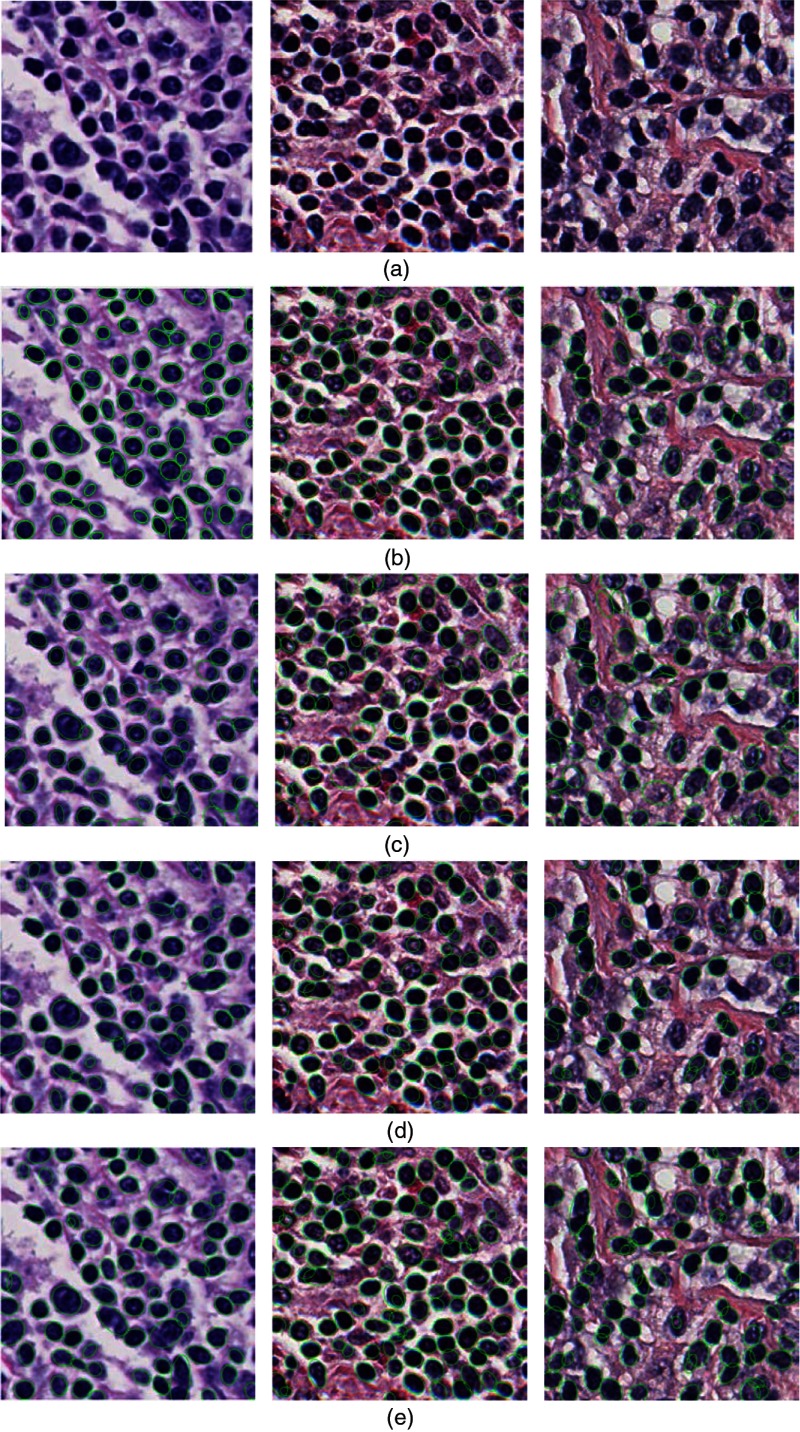
Examples of some segmentation results: (a) original H&E-stained image, (b) segmentation results with our proposed method, and (c) to (e) segmentation results with method in Refs. 5, 29, and 56, respectively.

Figure 7 shows some segmentation results with our proposed algorithm, from which it can be visually seen that our scheme can achieve reasonable nuclei extraction results. To show the robustness of our method, the segmentation algorithms presented in Refs. 5, 29, and 56 were used as comparisons. The nuclei segmentation results achieved from the method in Refs. 5, 29, and 56 are also given in Fig. 7, and it is noted that the method in Refs. 5, 29, and 56 produced more undersegmentation problems. These segmentation results were also quantitatively analyzed. Here, the Dice similarity coefficient (DCS), sensitivity (SN), and positive predictive value (PPV)^5^ were adopted as metrics for the segmentation evaluation. The DCS, which is a measure of overlap between two areas, is widely used as a segmentation evaluation and is defined as

$$\mathrm{DSC}(A_{\mathrm{seg}},A_{\mathrm{gt}})=2\frac{\left|A_{\mathrm{seg}}\cap A_{\mathrm{gt}}\right|}{\left|A_{\mathrm{seg}}|+|A_{\mathrm{gt}}\right|},$$ (12)

where $A_{\mathrm{seg}}$ and $A_{\mathrm{gt}}$ are the segmented region and the region of “ground truth” that are extracted by an expert pathologist and |•| means the number of pixel points in a certain region ($A_{\mathrm{seg}}$ or $A_{\mathrm{gt}}$). The DCS tends to be 1 when the segmentation results are very similar to the ground truth. For a segmentation approach, the value of the DCS is the higher, the better. The SN and PPV, which are metrics to evaluate target detection, are given as follows:

$$\mathrm{SN}=\frac{\mathrm{TP}}{\mathrm{TP}+\mathrm{FN}},\;\mathrm{PPV}=\frac{\mathrm{TP}}{\mathrm{TP}+\mathrm{FP}},$$ (13)

where TP is the true positive, which refers to the number of cell nuclei correctly detected; FP is the false positive, which represents the number of cell nuclei incorrectly detected; and the FN is the false negative, which denotes the number of cell nuclei undetected. Similar to the DCS, the higher the values for SN and PPV, the better the proposed segmentation algorithm. Here, a total of 10 images extracted from 10 randomly selected slide images are interactively segmented by a pathologist with the method present in Ref. 64, and these segmented cells are used as the ground truth for a metrics measurement. There are a total of 600 nuclei in the 10 extracted images. The method in Ref. 64 was chosen to get the ground truth nuclei because the segmentation results can be improved by interactively labeling more nuclei target regions and background regions based on segmentation results from the previous step until satisfactory results are achieved. Since the truthing process is time–consuming, the evaluation is only based on a set of randomly sampled data, which could be a potential limitation of this study. The results of the quantitative evaluation of our proposed segmentation algorithm and those from Refs. 5, 29, and 56 are given in Table 1. A t-test^65^ was conducted for all metrics between the proposed method and the other three approaches. All p values were smaller than 0.01, which means the difference is significance. Consequently, it can be seen from Table 1 that the performance of our proposed approach is superior to the other three methods. The main reason affecting the segmentation results in Ref. 5 may be the criterion to merge segmentation results under different image scales, while it is not easy to set the predefined threshold in Ref. 29 that will affect marker extraction and the final segmentation results. It is assumed in Ref. 56 that a one-to-one correspondence between the markers and objects exists. However, this will produce an under- or oversegmentation problem when the assumption is not satisfied. In fact, the assumption is not satisfied in many histopathological slide images because of the complex boundaries of the cell nuclei.

**Table 1:** Evaluation results of cell nuclei segmentation.

Metrics	DSC	SN	PPV
Method in Ref. 5	0.815	0.900	0.928
Method in Ref. 29	0.794	0.877	0.885
Method in Ref. 56	0.755	0.819	0.932
The proposed method	0.880	0.931	0.985

**Fig. 8:**
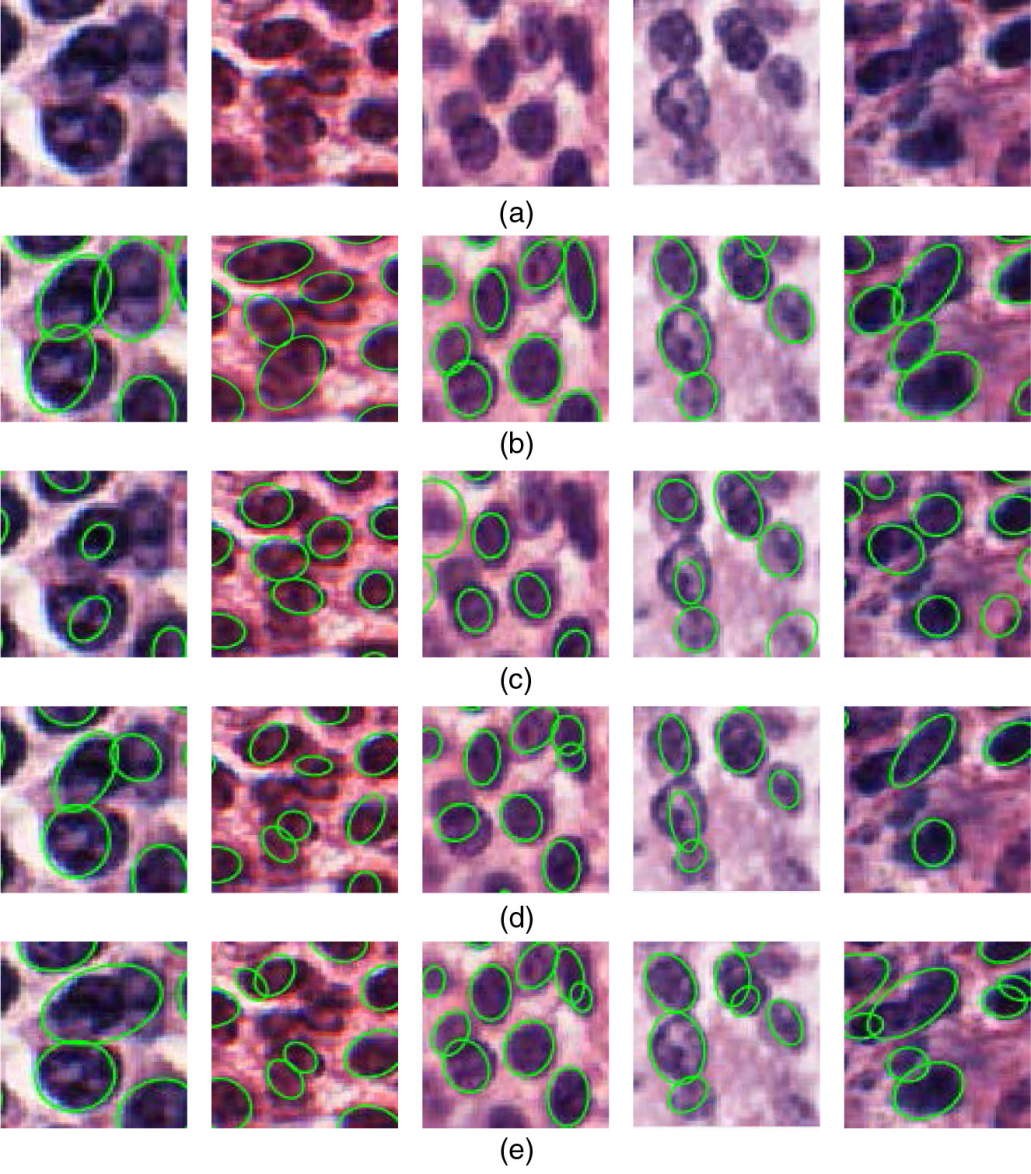
Separation of touching cell nuclei: (a) original H&E-stained image, (b) separation results with our proposed method, and (c) to (e) separation results with method in Refs. 5, 29, and 56, respectively.

The ability to separate touching nuclei is another element that needs to be evaluated in the nuclei segmentation algorithm. The segmentation results using our proposed methods and those in Refs. 5, 29, and 56 are presented in Fig. 8. It can be found that our method is robust enough to separate the touching nuclei. However, the methods in Refs. 5, 29, and 56 fail to separate some overlapping nuclei and produce an oversegmentation problem. Here, the underseparating, overseparating, and encroachment errors are used to quantitatively evaluate the performance of cell nuclei separation in the segmentation algorithm. Underseparating is defined as no splitting of the touching nuclei, whereas overseparating refers to separation within a single nontouching cell, and the encroachment error is described as an incorrect nucleus separation.^66^ A total of 224 regions having connected nuclei are used to statistically determine the predefined metrics. Table 2 shows the quantitative evaluation results. It can be found that our proposed method can achieve better nuclei separation in terms of underseparating, overseparating, and encroachment errors. It is not robust to detect the number of nuclei in touching nuclei regions in Refs. 5, 29, and 56. Consequently, it sometimes fails to separate a touching region or produces an oversegmentation problem.

**Table 2:** Evaluation results of cell nuclei separation.

Metrics	Undersplit	Oversplit	Encroachment error
Method in Ref. 5	0.112	0.025	0.161
Method in Ref. 29	0.192	0.029	0.138
Method in Ref. 56	0.143	0.056	0.143
The proposed method	0.076	0.022	0.098

A comparison of time consumption (TC) on a CPU for our proposed method and those in Refs. 5, 29, and 56 is listed in Table 3. These results are achieved from 10 images and each one has a size of 350×350. It can be noted that the TC of our method is on average higher than the approaches in Refs. 29 and 56 while it is less than the scheme in Ref. 5. This can be explained as the required time being strongly affected by the complexity of the algorithm, especially in the segmentation refinement part among these algorithms. For the methods in Refs. 29 and 56, the postprocessing step is only involved in the distance transform and morphological operation, and it is much simpler than our method and that in Ref. 5. However, the method in Ref. 5 is more complex than our algorithm because many cell features need to be measured and multiple scale images are processed. Even though our proposed algorithm is not the most efficient one in terms of TC, our method has better results in terms of segmentation accuracy and cell nuclei separation (see Tables 2 and 3).

**Table 3:** Evaluation results of consuming time (unit: s).

Image	Method in Ref. 5	Method in Ref. 29	Method in Ref. 56	The proposed method
Image 1	8.55	2.47	2.69	2.70
Image 2	7.33	2.58	2.97	3.83
Image 3	6.54	2.41	2.67	3.39
Image 4	8.50	2.58	2.88	3.85
Image 5	6.94	2.61	2.97	4.41
Image 6	9.37	2.67	2.91	4.45
Image 7	10.06	2.77	2.97	4.65
Image 8	10.34	2.68	2.98	4.10
Image 9	11.50	2.60	2.67	5.09
Image 10	9.54	2.55	2.59	3.99
Average	8.87	2.59	2.83	4.05

There are many parameters in our proposed algorithm, and some of them are hyperparameters that will affect the final segmentation results. Therefore, it is necessary to know more about these parameters. In Sec. 2.1, there is no variable that needs to be set manually. In Sec. 2.2, two kinds of morphological operations, which are opening by reconstruction and closing by reconstruction, are involved in the size of the structuring element. Usually, the size of the structuring element in this step should be similar to the size of the nuclei. However, the size of the nuclei is very flexible and not fixed, which increases the difficulty of selecting the size of the structuring element. We have conducted an SN analysis and the results are robust against choices of the size of the structuring element when it is around 7. In Sec. 2.3, there is no hyperparameter because the threshold value is automatically searched based on the image. In Sec. 2.4, the radius of the disk-shaped structuring element is assigned as 3, which would be used in the morphological opening operation so as to make the object boundary smooth. A small disk-shaped structuring element is enough to smooth the boundary, while a bigger structuring element would undermine the shape of the nuclei. Therefore, it is suggested to set this radius value to be 3. In addition, there is another parameter in Sec. 2.4, which is a threshold value. The threshold value is used to get the internal markers by combining with the H-maximum transform. This threshold value is set to be the small value 3 that will keep the internal marker having the nuclei’s shape. Even though a small threshold value can separate some slightly connected nuclei, it cannot dispatch other nuclei with more areas overlapping. However, this small threshold value will not affect the final segmentation results, because the nuclei separation is specifically done in the segmentation refinement part. In other words, the final result is not very sensitive to this threshold value when it is around 3. Moreover, the disk-shaped structuring element used in the morphological skeleton algorithm has the smallest size and is fixed. Therefore, it can be viewed as a constant. In Sec. 2.5, the number of clusters in the k-means algorithm is iteratively given and the proper one is selected once the objective function is minimized. That is, the value of this variable is automatically obtained. Additionally, all the parameters in Eq. (10) are measured from the segmentation results at the previous step. Thus, no parameter value should be manually given in advance. In other words, there are no parameters that need to be tuned in Sec. 2.5.

## 4 Conclusions and Future Work

In this paper, we developed an automatic method that is able to segment cell nuclei in H&E-stained histopathological images. The hematoxylin image that can appropriately represent the concentration of the hematoxylin stain is obtained with a color deconvolution algorithm, and all of the following processes are conducted based on the hematoxylin image. Morphological operations and automated thresholding techniques applied to the hematoxylin image make the segmentation result robust to the nuclei diversity and image heterogeneity while the segmentation results are further refined by minimizing a designed objective function so that the clustered cell nuclei can be separated. Our experimental results reveal that the proposed nuclei extraction approach can obtain good segmentation results and achieve better performance in terms of segmentation accuracy and nuclei separation compared with other nuclei extraction algorithms. We have tried different combinations of methods, but this particular series of image processing methods gives the best results among all other combinations. To the best of our knowledge, the segmentation refinement method is an idea that can determine the nuclei number within a connected region and separate the multiple nuclei. Even though our method has proven effective in separating touching nuclei, it still suffers from an oversegmentation problem when the nuclei shape is far from elliptical. Adding more information such as nucleus size into the objective function may be a way to solve this problem.

Based on the nuclei segmentation results, it may be fruitful to perform cell classification as a future work that would be beneficial to pathologists. One idea is to extract the cell features from the segmented cell nuclei and classify the cells by using traditional classification schemes. Another is to extract image patches centered with the segmented cell nuclei from the original H&E-stained histopathological image and learn the cell feature itself using a deep learning approach so that the researchers no longer need to define and analyze the features by themselves. The learned features may then be used for cell classification. Furthermore, the proposed segmentation algorithm may be helpful in cell tracking when a series of time-lapse images are available, and the cell tracking can be beneficial to the analysis of cell cycle progress and the understanding of drug effects on cancer cells. Based on our experiences, this methodology can be extended to other organs, such as breast and kidney, in the same pathology because almost all malignant cells have similar atypia in the nucleus from a pathologist’s view. It is worth noting that the current method was developed based on H&E-stained images with 40× magnification (which is usually considered relatively high resolution for H&E-stained images). When applying this method to lower resolution H&E-stained images such as 20× magnification the size of the structuring element used in the morphological operations needs to be adjusted accordingly. In addition, this proposed method is more suited to nuclei that have an ellipse-approximated shape. If the cell nuclei are not in an ellipse-approximated shape, a situation that is fairly uncommon, the proposed method may not work well.
